# Clinical Implications of Cytology Negative Pleural Effusion in Advanced Stage Epithelial Ovarian Cancer—Insights from a Tertiary Cancer Center in Northeast India

**DOI:** 10.1007/s13193-025-02232-9

**Published:** 2025-04-25

**Authors:** Debabrata Barmon, Mahendra Kumar, Karthik Chandra Bassetty, Upasana Baruah, Dimpy Begum, Mouchumee Bhattacharyya, P. S. Roy, Shiraj Ahmed

**Affiliations:** 1 Department of Gynecological Oncology, Dr. Bhubaneswar Borooah Cancer Institute, Guwahati, Assam 781016 India; 2 Department of Radiation Oncology, Dr. Bhubaneswar Borooah Cancer Institute, Guwahati, Assam 781016 India; 3 Department of Medical Oncology, Dr. Bhubaneswar Borooah Cancer Institute, Guwahati, , Assam 781016 India; 4 Department of Oncopathology, Dr. Bhubaneswar Borooah Cancer Institute, Guwahati, , Assam 781016 India

**Keywords:** Advanced staged ovarian cancer, Pleural effusion, Cytology negative pleural effusion, Thoracoscopy in ovarian cancer, Video-assisted thoracoscopic surgery

## Abstract

Pleural effusion is a decisive factor in advanced-stage ovarian cancer. The presence of malignant cells in pleural effusions in women with ovarian cancer is accepted as a poor prognostic factor. It is included in the International Federation of Gynecology and Obstetrics (FIGO) stage IVA. Still, the literature does not explain and prognoses the importance of cytology-negative pleural effusion (CNPE) in women with ovarian cancer. It is a retrospective study conducted in a tertiary cancer center. All advanced staged ovarian cancer patients with pleural effusion registered between January 2020 and December 2021 were included, and the control group consisted of all stage IIIC disease with no pleural effusion during the same duration. Survival analysis was done in these three groups—cytology-positive pleural effusion (CPPE), cytology-negative pleural effusion (CNPE), and no pleural effusion (NPE). In total, 117 patients with advanced-stage ovarian cancer, fulfilling the eligibility criteria, completed their treatment during the study period. Retrospective data was collected from hospital records, and survival analysis was done using SPSS 29.0. We included only those patients who had pleural fluid analyzed by a pathologist. During the study period, we found that 13 (11%) were CPPE, 23 (19.3%) were CNPE, and 81 (68%) were NPE. CNPE patients had poor progression-free survival (PFS) and overall survival (OS) compared to NPE patients, although both groups were labeled stage IIIC. These findings underscore the importance of cytology-negative pleural effusion in ovarian cancer prognosis, providing valuable insights for clinical practice. Patients with cytology-positive pleural effusion had the worst prognoses. However, CNPE patients labeled as stage IIIC had poor outcomes compared to NPE stage IIIC patients. So, based on our comprehensive study, we recommend a video thoracoscopic analysis of all patients with CNPE to correctly stage these patients, further modify their treatment accordingly, and improve their outcomes.

## Introduction

Ovarian cancer (OC) encompasses a heterogeneous group of malignancies, with the majority (90%) being epithelial ovarian cancers [1]. Less than half of patients with OC survive beyond 5 years after diagnosis because more than 70% of patients are diagnosed in an advanced stage [2, 3]. OC is unique because maximal surgical cytoreduction of all tumor sites increases survival [4]. Whether surgery in the primary setting or performed after neoadjuvant chemotherapy, the surgical goal should be to remove all gross disease altogether, leaving little (<1 cm; “optimal” cytoreduction) or no visible residual tumor [5].

In advanced-stage ovarian cancer, the presence of pleural effusion is a decisive factor [6]. The presence of malignant cells in pleural effusion in women with ovarian cancer is accepted as a poor prognostic factor and hence is included in the FIGO stage IVA [7]. Pleural fluid cytology is an excellent modality to diagnose malignant pleural effusion, but its sensitivity is poor. Diagnostic thoracoscopy can be used to assess the presence of pleural-based disease in CNPE cases for proper staging, although its role in malignant ovarian cancer is not well defined [8].

Usually, when patients are administered neoadjuvant chemotherapy in stage IVA and are operated on after 3–4 cycles of neoadjuvant chemotherapy (NACT), if pleural effusion resolves, the pleural cavity is not explored intraoperatively for residual disease [9]. Similarly, no study in the literature assesses the prognostic significance of negative pleural fluid cytology in OC. With this novelty, we planned this retrospective study to fill this gap in the existing knowledge.

### Study Objectives

To study the survival outcomes (PFS and OS) in cytology-negative pleural effusion (CNPE) in malignant ovarian cancer patients.

## Methodology

It is a retrospective study conducted at Dr. Bhubaneswar Borooah Cancer Institute, Guwahati (a unit of Tata Memorial Centre, Mumbai). After institutional research board (IRB) approval, patients were identified from institutional electronic medical records (EMR). All patients with a histologically confirmed diagnosis of advanced ovarian cancer (stage IIIC and IVA) who underwent treatment at Dr Bhubaneswar Borooah Cancer Institute from January 2020 to December 2021 were included in the study. Control groups were taken as stage IIIC disease with no pleural effusion. So, the study population was divided into three groups—cytology positive pleural effusion (CPPE) Stage IVA, cytology negative pleural effusion (CNPE) Stage IIIC, and Stage IIIC with no pleural effusion (NPE). At least one positive report was required for cytological malignant pleural effusion (CPPE) diagnosis. In contrast, a maximum of 3 times negative pleural effusion cytology report was required to label it as Stage III C with CNPE. We have labeled “round” to each times patient underwent pleural fluid cytology examination, like 1^st^ time cytology was termed as 1^st^ round of plural fluid cytology, 2^nd^ term cytology was termed as 2^nd^ round of cytology, and so on. The study did not include stage I, II, IIIA, and IIIB malignant epithelial ovarian cancer. Non-epithelial cancer, benign tumors of the ovary, other distant metastasis (stage IVB), recurrent malignant ovarian malignancy, and pregnancy were also excluded from the study group. After collection of demographic data including age, stage, histology, chemotherapy regimen, surgery performed, and recurrence of disease was noted, all patient received 3–4 cycles of paclitaxel and carboplatin (according to their performance status). After completion of neoadjuvant chemotherapy, the patient was evaluated for response to chemotherapy. Patients who have responded to chemotherapy underwent interval debulking surgery (IDS). Patient residual disease and cancer completion scores were noted. After 2–3 weeks of IDS, patients received 3 cycles of adjuvant chemotherapy of a regimen of paclitaxel and carboplatin. No patient in the study population received any maintenance chemotherapy. Patients were followed up for at least 24 months from completion of treatment. The patient’s last visit and disease status at the time of last contact were used to calculate survival. Progression-free survival and follow-up were calculated from the date of diagnosis of malignancy to the date of recurrence or last contact. Overall survival was also calculated from the date of diagnosis of malignancy to the date of death or last follow-up. Kaplan-Meier survival curves were generated for the various stages. Multivariate analysis was done for CPPE groups to know the relationship between positive pleural fluid cytology and overall survival on the statistical package for the social science (SPSS) 29.0. Progression-free and overall survival were calculated, and survival analysis was performed using SPSS 29.0.

### Ethical Clearance

The research protocol was approved by the Institutional Ethics Committee of the Dr. Bhubaneswar Borooah Cancer Institute, Guwahati, Assam, India.

## Results

**Fig. 1:**
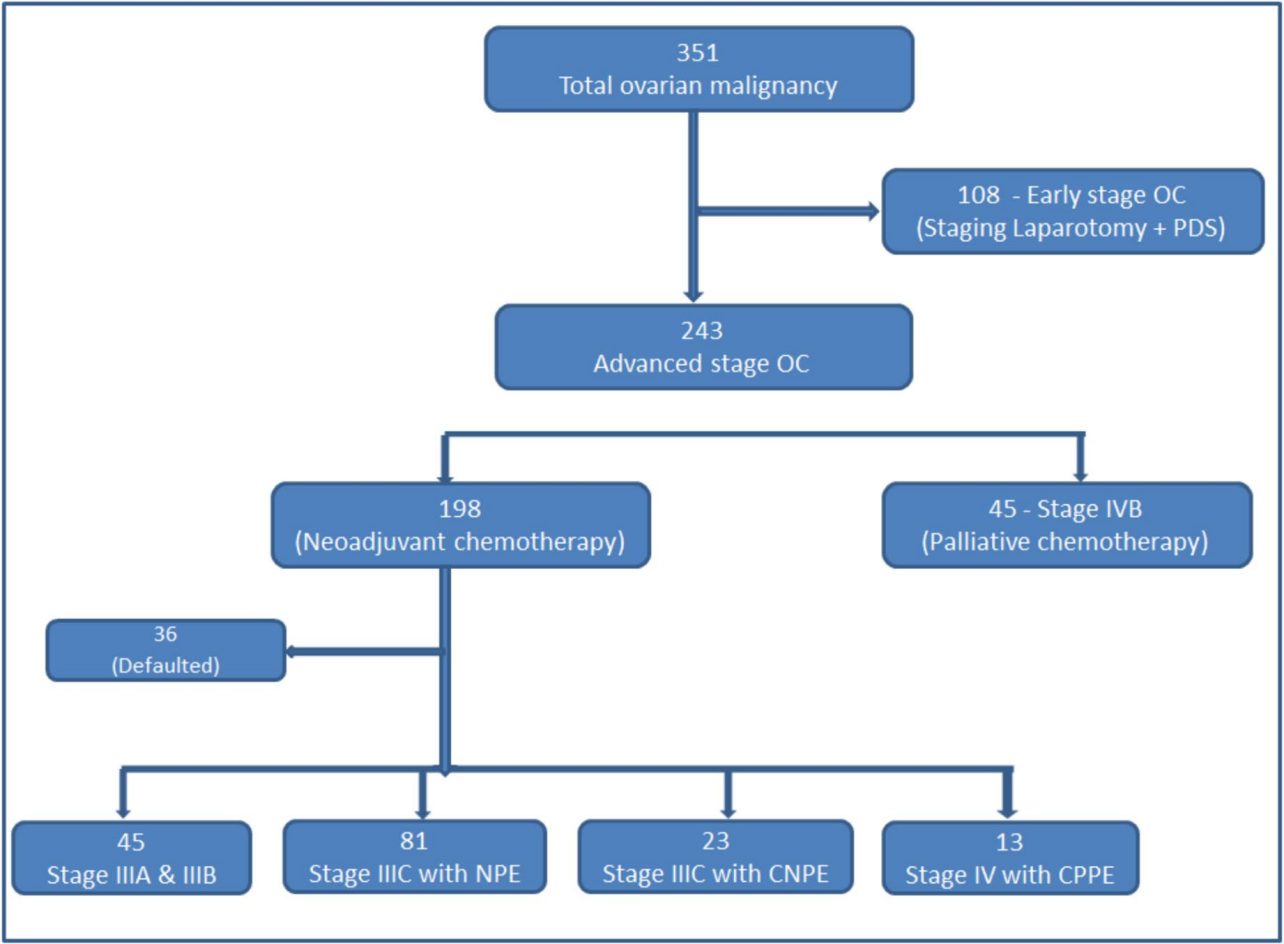
Distributions of registered ovarian cancer patients

During the study period, 351 patients of OC were registered in our hospital in the gynecological oncology department. A total of 108 patients underwent laparotomy for adnexal mass, and 243 patients were planned for chemotherapy. In the chemotherapy group patient, 45 patients were diagnosed with stage IVB ovarian cancer, most of which received chemotherapy with palliative intent; the rest of the 198 patients were planned for neo-adjuvant chemotherapy followed by interval debulking surgery. However, 36 patients defaulted or did not complete treatment in this institute. Of the remaining 162 patients, 45 belonged to IIIA and IIIB stages, with 81 patients in stage IIIC with NPE, 23 in Stage IIIC with CNPE, and 13 in stage IVA with CPPE. So, a total of 117 patients were included in the study. The distribution of these patients is shown in Fig. 1.

Table 1 shows the demographic profile of the patients. The median age of patients was 47 years. The majority of patients (70%) had parity greater than two. One-fourth of women were diabetic. Only high-grade serous carcinoma patients were included in our study.

**Table 1:** Demographic profile

S. No	Category	*N* = 117 (%)
1	Median age (range)	47 years (22–74 years)
2	Parity	
	i. Nulliparous	21 (18%)
	ii. ≤ 2	43 (36%)
	iii. > 2	53 (46%)
3	Comorbidities	
	i. Diabetes mellitus	31 (27%)
	ii. Hypertension	22 (18%)
	iii. Others	4 (2.7%)
	iii. No comorbidity	60 (52.3%)
4	FIGO stage	
	i. IIIC without pleural effusion	81
	ii. IIIC with CNPE	23
	iii. IVA (CPPE)	13
5	Histology	
	HGOC	117 (100%)
6	PCI (peritoneal cancer index)	
	1. < 10	45 (38.4%)
	2. 10–20	54 (46.1%)
	3. > 20	9 (7.6%)
7	CC score (completeness of cytoreduction score)	
	1. 0	54 (46.1%)
	2. 1	51 (43.8%)
	3. 2	10 (8.5%)
	4. 3	2 (1.7%)

Out of the total of 117 patients in the study group, 36 patients had pleural effusion. In first round of plural fluid cytology, all patients underwent pleural fluid cytology for malignant cells. After the first round of pleural fluid cytology, nine patients were reported as positive cytology, whereas 27 were cytology negative. In the second round, remaining 27 patients underwent pleural fluid cytology, in which 3 new patients were diagnosed as pleural fluid cytology positive for malignant cells. Finally, in third round of pleural fluid cytology, 24 patients underwent cytology examination, and 1 more patient reported as positive for malignant cells. So, total 13 patients labeled as CPPE and remaining 23 patients were labeled as CNPE (Table 2), and further management was done according to the set protocol of our institute.

**Table 2:** Results of pleural fluid cytology reports in each round

No	Rounds	Cytology positive	Cytology negative
1	1st round	9	27
2	2nd round	11	25
3	3rd round	10	26

Finally, all diagnosed patients of pleural effusion were symptomatically managed. Out of 36 patients, 25 patients were required some therapeutic intervention. In our study population, 9 patients of CPPE groups, and 16 patients of CNPE group required some kind of intervention. Intervention was either intercostal drainage (ICD) insertion or therapeutic pleurocentesis. Out of 25 patients, 18 patients (72%) were required therapeutic pleurocentesis, whereas only 7 patients (28%) required intercostal drainage.

Multivariate analysis was performed using SPSS 29.0 to correlate overall survival with fluid cytology results (Table 3). The result of the pleural fluid cytology report of positive (presence of malignant cell) was affecting the overall survival of patients (*P* value 0.001). Pleural fluid cytology was negatively correlated (− 0.63) with the overall survival of patients, which means that as the positivity of pleural fluid cytology increases, the chances of overall survival decrease.

**Table 3:** Multivariate analysis between rounds of pleural fluid cytology and overall survival

Source	Dependent variable	Type III sum of squares	df	Mean square	Sig
Corrected model	Round 1	2.769^a^	11	.252	< .001
	Round 2	1.692^a^	11	.154	< .001
	Round 3	1.692^a^	11	.154	< .001
Intercept	Round 1	5.565	1	5.565	< .001
	Round 2	8.696	1	8.696	< .001
	Round 3	8.696	1	8.696	< .001
OS	Round 1	2.769	11	.252	< .001
	Round 2	1.692	11	.154	< .001
	Round 3	1.692	11	.154	< .001
Error	Round 1	4.468E − 31	1	4.468E − 31	
	Round 2	4.450E − 30	1	4.450E − 30	
	Round 3	9.861E − 32	1	9.861E − 32	
Total	Round 1	9.000	13		
	Round 2	11.000	13		
	Round 3	10.000	13		
Corrected total	Round 1	2.769	12		
	Round 2	1.692	12		
	Round 3	1.692	12		

Along with 81 patients with stage IIIC with NPE, these patients (36 patients) received their treatment (chemotherapy with surgery followed by adjuvant chemotherapy). After adjuvant chemotherapy was completed, patients were followed up regularly. The progression-free survival period was calculated from the completion of primary treatment to the occurrence of the first recurrence or the last follow-up. All recurrent patients during this period were undergoing treatment at our institute. Finally, the overall survival period was calculated from the completion of primary treatment to the patient’s death or the last follow-up. Any patient who was lost to follow-up was censored after that period. PFS and OS were calculated, and an analysis was performed using SPSS 29.0 software.

**Fig. 2:**
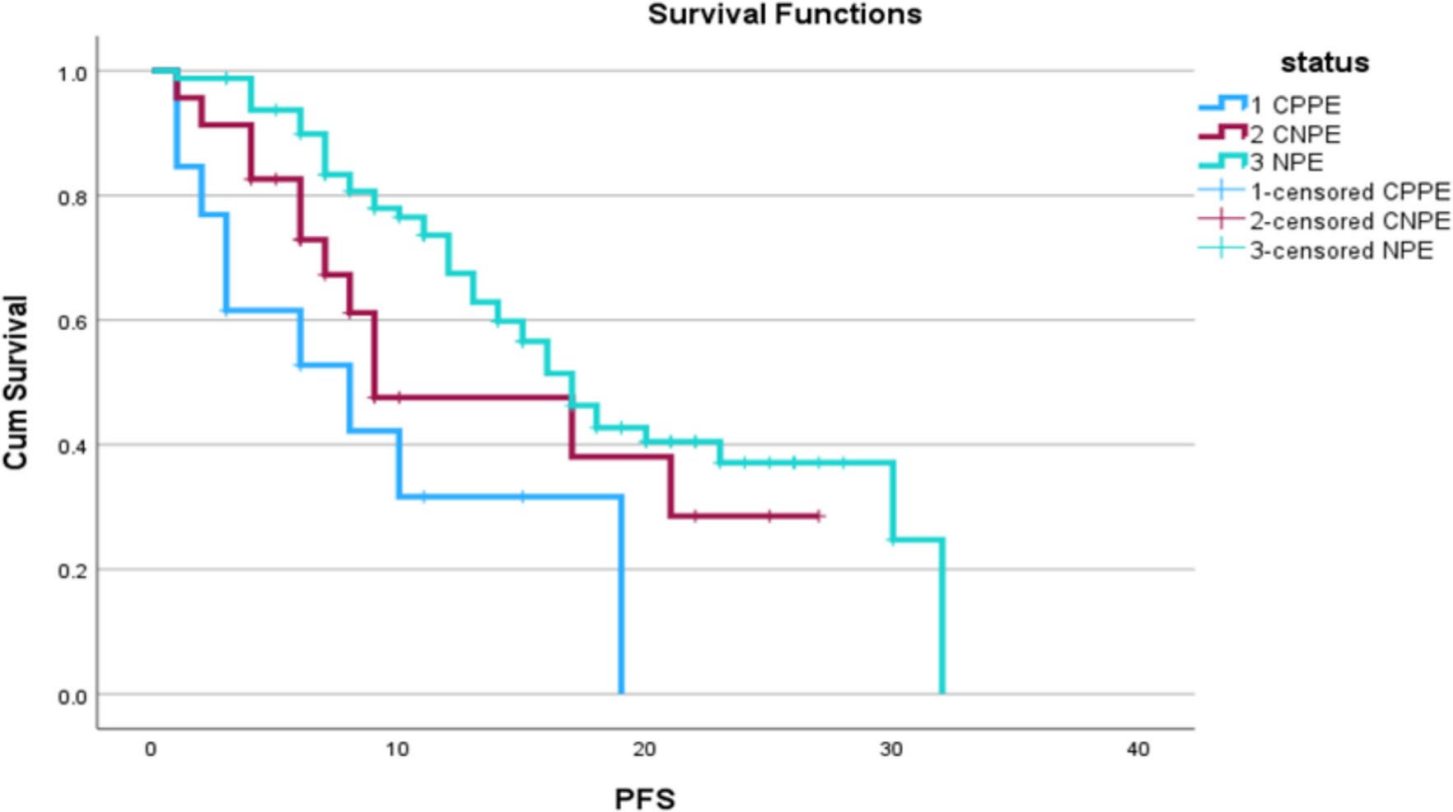
Kaplan–Meier curve showing CPPE, CNPE, and NPE group PFS

**Fig. 3:**
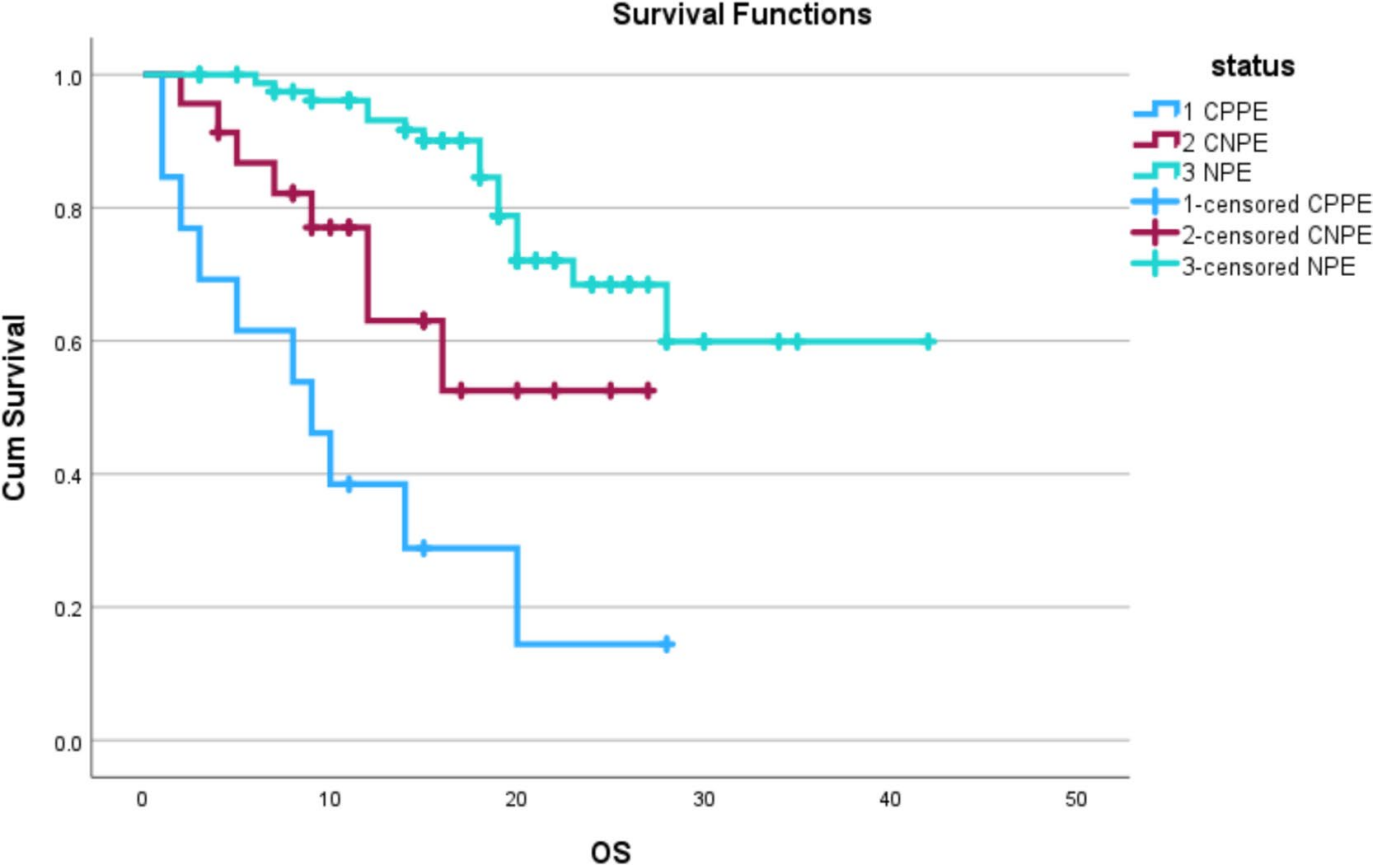
Kaplan–Meier curve showing CPPE, CNPE, and NPE group OS

These patients were followed up for a median period of 27 months. When analyzed in the Kaplan–Meier curve for progression-free survival, as shown in Figs. 2 and 3, patients with CPPE had worse than those with CNPE and NPE (*P* value < 0.05). There were 42 (51.8%), 12 (52.1%), and 9 (70%) recurrences in the NPE, CNPE, and CPPE groups, with median PFS of 17, 9, and 8 months (statistically significant with *P* value < 0.05).

Similarly, patients with CPPE had poor OS compared to CNPE and NPE. There were 18 (22.2%), 8 (34.7%), and 10 (77%) deaths in NPE, CNPE, and CPPE groups, with the mean OS of 32, 18, and 11 months (statistically significant with *P* value < 0.05).

## Discussion

According to NCCN guidelines, the majority of advanced-stage ovarian cancer patients (Stage IIIC and IVA) are managed with neoadjuvant chemotherapy followed by interval debulking surgery and adjuvant chemotherapy [9]. Interval debulking surgery (IDS) is performed after neoadjuvant chemotherapy after assessment of the response to chemotherapy. In complete response and partial response patients, IDS is performed; disease progression will require further evaluation and a change of chemotherapy, whereas stable disease can undergo IDS or further chemotherapy depending on the surgeon’s choice [10].

Complete or optimal cytoreduction during primary or interval cytoreduction surgery is an important prognostic factor for patient survival. The presence of residual disease after IDS results in poor outcomes in terms of PFS and OS [5].

With this background in mind, pleural effusion in ovarian cancer needs proper evaluation before treatment. Pleural effusion is thought to be present in more than one-third of individuals with Stage IV ovarian cancer. The presence of malignant pleural effusion before chemotherapy requires proper evaluation before IDS to rule out residual disease in the pleura. The macroscopic intrathoracic disease may change the patient’s therapy, particularly if unresected > 1 to 2 cm intrathoracic tumor deposits would result in unsatisfactory residual disease at the end of maximum intra-abdominal cytoreduction. The VATS (video-assisted thoracoscopic surgery) can detect the pleural tumor burden, allow intrathoracic cytoreduction, and sometimes reveal gross tumor residue in the pleural cavity, indicating that abdominal surgery is unnecessary [11, 12].

Cytological analysis of pleural fluid is often the first-line diagnostic test after detecting an effusion. Thoraco-centesis to obtain a fluid sample is a simple bedside procedure with low complication rates [13]. It is, therefore, more accessible and cost-effective than potentially higher yield but more invasive methods such as thoracoscopy. Over the last three decades, international literature has described a 40–90% sensitivity of pleural fluid cytology for detecting malignant pleural effusion (MPE) in various locations and clinical settings [13–20]. The diagnostic yield of fluid cytology is relatively low (averaging 60%) and varies considerably by tumor type [21]. The negative predictive value of pleural fluid cytology is low. Despite the absence of malignant pleural effusion after pleural fluid cytology, there is the possibility of hidden malignant cells or disease.

Current pleural disease guidelines advocate a stratified diagnostic approach to MPE, whereby thoracoscopy is only offered if fluid cytology is negative (i.e., no malignant cells are seen) [13]. Eisenkop investigated the predicted advantages of thoracoscopies as a treatment strategy for Stage IIIC-IV epithelial ovarian cancer by performing VATS simultaneously with primary cytoreduction in 30 patients for detecting intrathoracic disease and the possibility of cytoreduction [22]. The survival rates for patients categorized in Stage IV undergoing a thoracoscopy were found to be longer than the ones who did not.

The 5-year survival rates of patients with stage III and IV ovarian cancer were 36% and 17%, whereas the 10-year survival rates were 23% and 8%, respectively [23]. The 8-year survival rates of patients with stage I/II and III/IV ovarian cancer were 85.7% and 20.0%, respectively [24].

The 5‐year survival rate of advanced-stage ovarian cancer was reported to be 18–30% [25]. The above finding is similar to our study.

The study’s strengths are its novelty, rigorous methodology, statistical analysis, and ethical considerations. In our research, we have calculated the widely accepted survival analysis progression-free survival and overall survival. Ethical considerations and clinical implications also strengthen our study. A retrospective design is a fundamental weakness of our study. Along with a single-center study, the sample size was unequal and limited to particular stages of the disease, which was also a weak part of our study. Due to its retrospective nature, sampling and data collection biases are inevitable. We propose to conduct a prospective multicenter trial to validate the findings. We also propose introducing newer techniques like thoracoscopy to diagnose pleural-based disease and newer biomarkers to diagnose pleural fluid malignancy.

## Conclusion

Pleural effusion is a poor prognostic factor in ovarian cancer, irrespective of the presence of malignant cells in pleural cytology. The worst prognosis was seen in patients with cytology-positive pleural effusion (CPPE). We recommend thoracoscopic assessment of patients with pleural effusion in initial diagnostic evaluation, especially in cytology-negative pleural effusion. Further prospective studies must be carried out to better manage this group of patients.
